# Inspection of Dental Offices by the State Board of Health

**Published:** 1914-12-15

**Authors:** 


### INSPECTION OF DENTAL OFFICES BY THE STATE BOARD OF HEALTH.

Recent amendments to the sanitary code of the State of Louisiana provide for the inspection of the offices of dentists, physicians, etc., by the State Board of Health.

The amendment states that hereafter all offices, sanitoria parlors and other places, whether in charge of a physician, dentist, dermatologist or other person treating or in any wise attempting to cure any human ailment, shall be subject to inspection by this board.

Such offices shall be scored for points and according to the model score card, and when such offices shall fall below fifty points upon such inspection and scoring, the board will cause to be made against the person primarily responsible for the conduct of such office, charges for the infraction of this code.

To give the reader an idea of what the inspection consists, and the number of points given as perfect for the different items, we quote as follows:

Equipment—Location (2), surroundings (2).

Arrangement—Necessary rooms (1) conveniences (1).

Construction—Floor (2), walls (1), ceiling (1), light (1), ventilation (2), screens, fans (2).

Water—Hydrant (3), stationary (1), hot, abundance, (5), cool, abundance, (5).

Drinking Water and Glass—In cooler (2), otherwise (1).

Instruments—(4).

Library—(4).

Furniture—(4).

Journals—National (2), state (1), for three others (2).

Cleanliness—Floor (3), furniture (2), walls (2), ceiling (1), doors (1), windows (1), good condition, otherwise, (2), free from flies (2), free from bad odor (1).

Instruments—(4), tables, operating, (2), chairs, operating, (2), sterilizers (2), sinks and lavatory (3), toilets (3), disposition of old dressings, etc., (3).

Personal Appearance—Of attendant (2).

Dentist, Physician or Operator—Cleanliness of hands and finger nails (4), personal appearance and breath (6), general health (4).

At first thought this might seem like an uncalled-for imposition, but further reflection reminds us that if there are offices of dentists or physicians that are unsanitary, they should be made sanitary. Dentists and physicians are eternally preaching and advocating *cleanliness* and hygiene and they should put it into practice in person and office. It is one of the first duties that they owe their patients and themselves.

They need to take to heart the lesson of personal cleanliness and hygiene which they have been preaching to the layman, or the pupil will outstrip the master in the very things that the latter is teaching.

No dentist should have fear of an inspection by any board of health should the enforcement of such a code become general; on the other hand, he should at all times keep his office and person in such a presentable condition that it will afford him pleasure to have people come to his office for inspection or otherwise. In these days when the most modern and sanitary equipment can be obtained, there is no excuse for any dentist to have an office in such a condition that it requires the aid of a board of health to make it a fit place for patients.

Since writing this editorial a prominent business man of Detroit told the editor of an experience he had recently in a dental office, and having a direct bearing on this subject we are going to relate it to our readers:

This man said:

"Knowing the sincere and persistent efforts of leading men of your profession to purge it of its detriments and promote it to its highest degree of perfection and standing, I desire to call your attention to an unpleasant experience I recently had, in the hope it may lead to remedial legislation or enforcement of such statutes as relate to such a condition, should there be such laws.

"It happened in a small town where I was unacquainted. I had time between trains to spare, and I regarded it as an opportunity to have my teeth cleaned. I asked the barber who was the best dentist. He said they were all good. I asked which was the most recent graduate. I reasoned such a dentist would have the newest, most up-to-date apparatus and practice the most hygienic and aseptic methods. The entrance to the building was unsightly, and the dust and dirt were thick on the stairs to the second floor, where the most recent graduate had his establishment. It consisted of two rooms— one for reception, the other for work.

"He remained seated when I entered, but arose when I told him what I desired. Then I noticed his shirt protruding from the space between his belt and vest, very much like a stevedore. His shoes were unpolished, his trousers bagged at the knees, his collar soiled, and his necktie disarranged, while his hair was long and unkempt. All of these so-called personal detriments I quickly noticed, and more especially because he was a young man, first starting.

"He said I could take the chair in the adjoining room, which I did. Then the great need of some sort of dental inspection under local, state or government supervision was impressed upon me at each successive thing I looked upon. He had his working tools on an upright tray, and they were very old, which in itself is not cause for severe criticism, but they were corroded with chemicals and rust as a result of uncleanliness. The basin of the fountain cuspidor was littered with cotton sponges and stained with blood, which gave off a puss-like hue. He told me the plumber had'nt been around to fix it, and I honestly replied it looked like the bottom of a garbage can. He wore no coat; he hardly washed his hands. His work was like his equipment and personal appearance—rotten and on a level with a cuspidor in his reception room, almost overflowing with tobacco juice. Until I saw all these things, I had'nt noticed certain blemishes on his face and neck, and you may rest assured I lost no time finding a drug store, and returning to my hotel where I used up the largest portion of a bottle of antiseptic, until my mouth felt clean again.

"The whole affair was nauseating—dirt and filth every where. Isn't it a sad commentary *in these days* of warfare against contagion? The question arises: Should such an establishment be permitted to continue to exist? Can't the public be protected? Would we want to go back to the common drinking cup in public places; but isn't this young dentist as dangerous a medium of contamination?

"We have many good laws restricting and regulating matters pertaining to the public health, and it may be we have some to cover the case in question; I hope we have, and link this hope to another, that, if so, they will be enforced and the public safeguarded against such a detriment to the high standards established and being maintained by the leading men of your profession."

We had no idea that in these enlightened days such a state of things existed anywhere, but if this is only one of many similar offices it seems more reasonable than we first supposed that a State Board of Health should inspect dental offices.—*Western Dental Journal.*

